# Integrated TTF and self-determination theories in higher education: The role of actual use of the massive open online courses

**DOI:** 10.3389/fpsyg.2023.1108325

**Published:** 2023-02-02

**Authors:** Uthman Alturki, Ahmed Aldraiweesh

**Affiliations:** Educational Technology Department, College of Education, King Saud University, Riyadh, Saudi Arabia

**Keywords:** perceived relatedness, DMOOCs, self-determination theory, TTF theory, behavioral intention to use the MOOCs system, user satisfaction

## Abstract

The purpose of this study was to examine the relationships between users’ satisfaction with actual use of massive open online courses (MOOCs) and intrinsically motivated, task-technology fit, attitudes toward using MOOCs, and behavioral intention to use MOOCs. As the primary technique for data collection, a survey questionnaire on self-determination theory (SDT) as well as task-technology fit (TTF) was distributed to a total of 228 students. The results of the users’ (TTF) to attitude toward using MOOCs and their behavioral intention to use MOOCs had a positive impact on their satisfaction and actual use of MOOCs in higher education institutes. However, the users’ perceived autonomy was not entirely satisfied, based on the results of their intrinsic motivation for the actual use of learning courses. Similarly, technology characteristics were insignificant with TTF for the actual use of MOOCs in academic institutions. Additionally, mediation studies showed that the correlations between independent factors on the one hand and users’ satisfaction with their actual use of MOOCs on the other were significantly mediated by intrinsic motivation, TTF attitude, and behavioral intention to use. Finally, practical ramifications were examined, and recommendations were made with regards to the direction of future studies.

## Introduction

The use of mobile technology to deliver education to everyone has grown in popularity over the past several years (Lizcano et al., 2020). Online courses that a student accesses through the internet are a massive open online course (MOOCs). Typically, these courses consist of traditional class materials made accessible online, which may include the following: such as video lectures that have been filmed or recorded, interaction with other students *via* forums, online quizzes and examinations; and interactive learning modules (Amit et al., 2022). The opportunity to access world class education and lifetime learning may be offered to students through the use of MOOCs (Al-Rahmi et al., 2018). Millions of Internet users have enrolled in digital learning courses provided by MOOC platforms over the past few years, making MOOCs a well-liked learning platform (Al-Rahmi et al., 2021c), and one which is popular among learners (Jansen et al., 2022; Usher and Hershkovitz, 2022). World-wide access to MOOCs is unlimited (Ogunyemi et al., 2022). Open enrolment, content sharing, and adjustable outcomes are all possible in MOOCs. Massive open online courses (MOOCs) provide publicly interactive, free, and open virtual resources that are backed by leading specialists in the field. According to Goopio and Cheung (2021), student persistence in following MOOCs programs is influenced by a variety of factors, including carelessly thought-out course design, a lack of interaction, and limited student experience.

Furthermore, the success of MOOCs depends on the dedication of the students who take part, which is based on their learning goals, their prior knowledge and skills, and their shared advantages (Tao et al., 2022). College or universities cannot ignore the impact of MOOCs, even after considering the advantages and potential drawbacks of MOOCs (Dalipi et al., 2018; Al-Mekhlafi et al., 2022; Tao et al., 2022). The viability and benefits of MOOCs must therefore be thoroughly investigated in terms of the delivery of educational curriculum. According to Dalipi et al. (2018), learner-related factors such as a lack of enthusiasm, a lack of time, a lack of background knowledge and abilities, and MOOC-related factors are the two key aspects that predict learner dropout in the case of MOOCs (e.g., course design, feelings of isolation and the lack of interactivity, hidden costs).The usefulness of MOOCs has come under scrutiny because of their high dropout rates (Xing and Du, 2019; Al-Rahmi et al., 2021c), an aspect which is a source of worry for education experts (Moafa et al., 2018; Aldowah et al., 2020).

Numerous studies (Dalipi et al., 2018; Goopio and Cheung, 2021; Ghai and Tandon, 2022; Zaremohzzabieh et al., 2022) have been undertaken to determine the causes of attrition and lowering of retention rates in the case of MOOCs and for possible solutions. Moreover, Previous studies (Watted and Barak, 2018; Abdullatif and Velázquez-Iturbide, 2020; Maya-Jariego et al., 2020; Chaveesuk et al., 2022) focused on a certain type of motivation in relation to MOOCs dropout. According to a review of the literature on MOOCs, most studies in this area have been quantitative in nature (Zhu et al., 2020). According to a study of the literature, enthusiasm is one of the key elements that can affect students’ persistence in terms of MOOCs (Watted and Barak, 2018; Abdullatif and Velázquez-Iturbide, 2020; Maya-Jariego et al., 2020). Participants in MOOCs were divided into three groups by Maya-Jariego et al. (2020) based on their internal motivation, external motivation, and persistence. The authors continued by stating that motivational intensity is linked favorably to MOOCs engagement and satisfaction. According to Abdullatif and Velázquez-Iturbide (2020), Al-Rahmi et al. (2021a), and Sayaf et al. (2022), internal motivation substantially influences the intention to continue using digital learning tools (Sayaf et al., 2022) and that desire has a significant role to play in understanding learners’ behavior in the case of MOOCs.

The findings of a study by Chaw and Tang (2019) also supported the hypothesis that in MOOCs, there is a strong correlation between participant motivation and course completion. Although motivation plays a role in MOOCs dropout rates, it is still not apparent what kinds of motivational elements encourage students to complete MOOCs (Mohan et al., 2020). Moreover, a thorough analysis of motivational impact on MOOC dropout rates is required, according to academics (Wei et al., 2023).The factors that influence learners’ attitudes toward adopting MOOCs have also been studied using a variety of technology and innovation acceptance models, including the unified theory and acceptance and use of technology (UTAUT), theory of planned behavior (TPB), technology acceptance model (TAM), task technology fit (TTF), the technology organization-environment (TOE) framework, SDT, and social motivation. Based on a TUE viewpoint, Ma and Lee (2019) looked at the motivations for and obstacles to the uptake of MOOCs. The most important elements influencing MOOC uptake, according to the authors, are technological aspects of MOOCs such perceived usefulness, accessibility, and performance-to-cost value. SDT was used by Khan et al. (2018) to analyze student adoption of MOOCs. They discovered that perceived connectedness and perceived competence had a significant beneficial impact on the students’ inclination to use MOOCs. The antecedents of students’ participation in MOOCs were investigated by Sun et al. (2019) using SDT and the notion of relationship quality as guides. According to the authors, students’ intrinsic motivation, which boosts their psychological involvement in MOOCs, is significantly positively impacted when their psychological requirements for autonomy, competence, and relatedness are met. In order to study the variables influencing students’ decisions to engage with MOOCs, Luo et al. (2021) merged the TPB and SDT. The authors discovered that perceptions of behavioral control and attitudes about MOOCs were important determinants of students’ behavior intention with regard to using MOOCs.

To explain the determinants impacting MOOC uptake, Altalhi (2021) established a framework based on UTAUT principles, language abilities, and learner characteristics. Altalhi (2021) expanded the UTAUT with value to better analyze how students use MOOCs. Their model could only account for 49% of the variance in the behavior intent to enroll in MOOCs, which suggests that more research is necessary. The advantages include promoting the university’s national goals, which, in the case of Saudi universities are connected to digital learning courses and web-supported core skills, as well as the development of the university’s international student recruitment efforts. This study details the implementation of MOOCs at King Saud University in Saudi Arabia as part of the academic program, and aims to provide guidance for further MOOCs integration in Saudi Arabia higher education. Therefore, by examining the relationships between both the uniqueness of SDT factors and TTF in a similar model, this research supports the literature on TTF.

According to the research, SDT can be used as a situational theory to examine how inspirational elements affect TTF conceptions. This involves six characteristics: Perceived Relatedness, Perceived Competence, Perceived Autonomy, Perceived Value, Task Characteristics, Technology Characteristics as determinants of Intrinsic Motivation, Task-Technology Fit, and attitude toward using the MOOCs system, the Behavioral intention to use the MOOCs system, User Satisfaction, and Actual use of MOOCs system. The empirical investigation may aid in the development and testing of theories about the recognition of MOOCs, as well as the acceptance of designs for MOOCs systems on the part of practitioners. This study will combine the SDT paradigm and the TTF model to create an optimum conceptual model in order to precisely identify elements and the mechanisms that influence university students’ on going intention to use MOOCs. We attempt to respond to two research questions: What are the key elements influencing university users’ satisfaction and actual use of MOOCs? What are the individual and combined effects of SDT and TTF on college students’ satisfaction and actual utilization of MOOCs?

## Theoretical model and hypotheses

**Figure 1:**
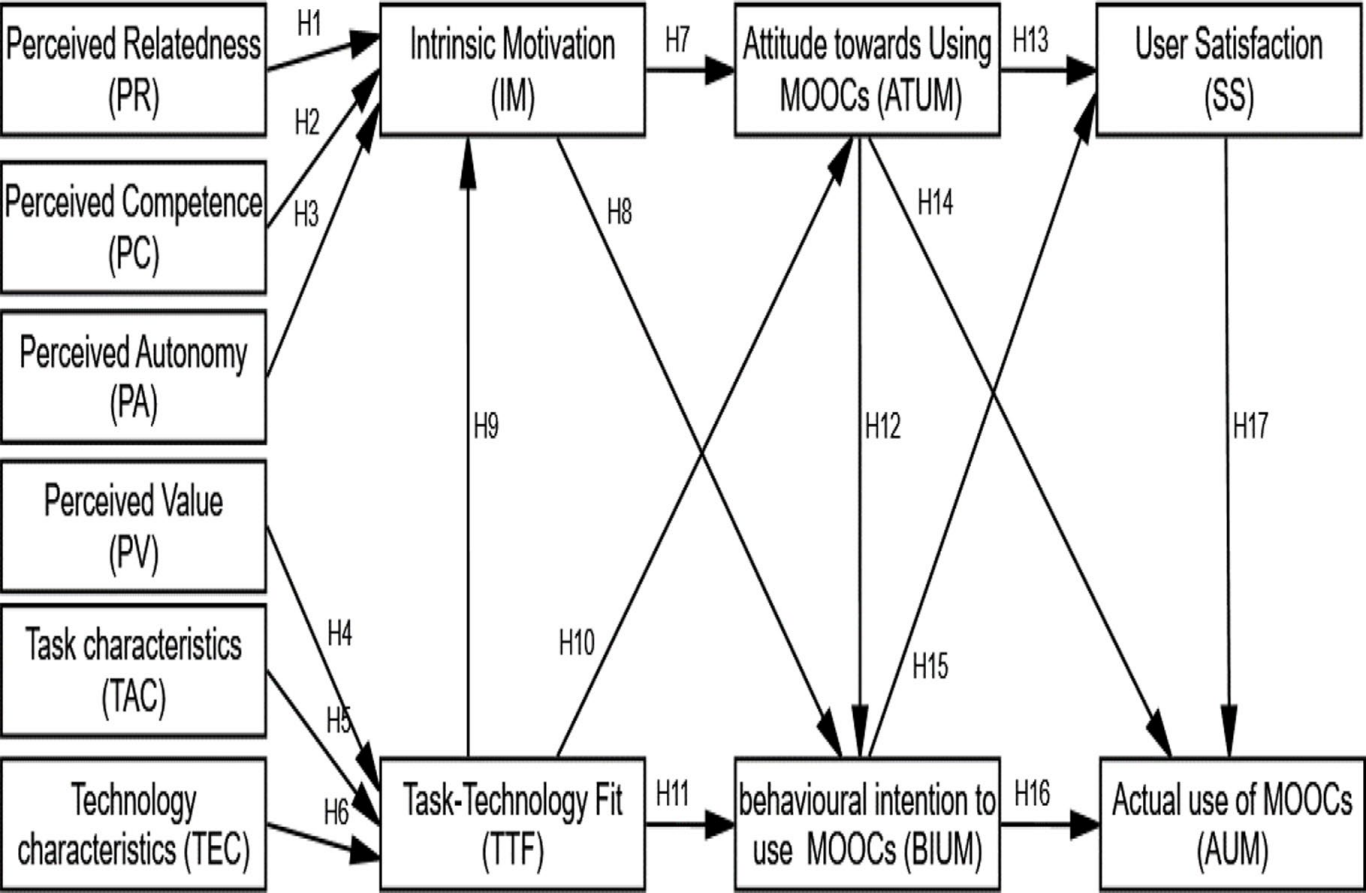
Research model.

In some situations, SDT and TTF are similar, and they work best together to monitor IS/IT deployment. According to academics, the SDT hypothesis represents a subset of perceived innovation traits; thus, the bringing together of these double theories could provide a more robust model than any single model could. Previous studies that have blended the two hypotheses have great results (Al-Rahmi et al., 2021b). As a result, the SDT and TTF are the two primary theoretical models used in this study. Leading theories, empirical research, and a survey of the literature on technological acceptability were then combined. Then, a model was then put forward that combines critical paradigms, including acceptance, attitude toward using MOOCs, user satisfaction, and the actual use of MOOCs (see Figure 1).

## Hypotheses development

### Self-determination theory

According to SDT, relatedness, independence, and competence are the three psychological factors that account for an individual’s intrinsic drive when it comes to adopting an innovation (Deci and Ryan, 2012).

#### Perceived relatedness

The desire of users to “interact with, be linked to, and feel caring for other people” is described as felt relatedness (Deci and Ryan, 2012). People are encouraged to utilize a certain technology because of feelings relatedness, which gives them the option to connect with others for mutual gain (Sun et al., 2019). Relatedness was discovered by Khan et al. (2018) to be a major predictor of MOOCs utilization intentions. Learners have the opportunity to connect with prominent academics, practitioners, and other scholars while pursuing their studies through MOOCs. These connections provide learners with a sense of pride, which motivates them to study *via* MOOCs (Sun et al., 2019). According to SDT, individuals are likely to be more supportive of their group’s goal when they feel connected to group members. Individuals still perform some uninterested or unenjoyable activities because they are valued by relevant others (e.g., peers, friends, superiors or institutions; Deci and Ryan, 2012). The value of MOOCs is also linked to the satisfaction of the demand for relatedness because relationships made with others can be advantageous for students in a variety of ways. Hence, the following hypothesis is formulated:

*H1*: PR has a positive effect on the MI.

#### Perceived competence

Perceived Competence displays people’s positive to feel successful and productive when performing a task (Deci and Ryan, 2012). By completing assignments and responding to tests, students can demonstrate their ability to other participants in a MOOCs environment. Their trust in learning is increased when their need to demonstrate competence is met, which pushes them to participate in MOOCs (Sun et al., 2019). MOOCs participation is encouraged by learners’ beliefs that completing a course will allow them to demonstrate their skills (Sun et al., 2019). Deci and Ryan (2012) assumed that satisfying the need of competence influence the level of motivation. Students who are satisfied with online SRL competence will feel qualified to regulate their own engagement in online learning. This feeling of being qualified is expected to be related with intrinsic and extrinsic motivation. Hence, the following hypothesis is formulated:

*H2*: PC has a positive effect on the MI.

#### Perceived autonomy

The concept of autonomy is the perception of independence when utilizing any system (Deci and Ryan, 2012). Perceived autonomy in the setting of MOOCs was described by Khalid et al. (2021) as the degree to which students believe they have the freedom to make their own decisions. According to Luo et al. (2021), MOOCs give students the opportunity to learn at their individual speed. The autonomy in terms of managing a particular course to meet the needs of the students fosters intrinsic motivation for MOOC-based learning (Luo et al., 2021). Further supported the idea that learners are motivated to use MOOCs when their demand for autonomy is satisfied. Indeed, several studies posited that autonomy promotes intrinsic and extrinsic motivation, which in turn produces positive outcomes. Khalid et al. (2021) showed that students present more internalization of the values in a course when they have more support with autonomy. Hence, the following hypothesis is formulated:

*H3*: PA has a positive effect on the MI.

#### Perceived value

The trade-off between the advantages of an innovation and the effort, risk, and financial costs incurred to bring it about is referred to as perceived value (Dearing, 2021). In this scenario, learners may question if it is worthwhile to invest time and money in MOOCs. They could contrast the advantages of using MOOCs for learning with the work needed to complete them. The only way for students to adopt MOOCs is if they believe it is advantageous to invest time, effort and money in them. According to Ma and Lee (2019), students are more likely to embrace MOOCs if the advantages of doing so outweigh the disadvantages. Utilitarian value reflects an aspect of perceived value (Watjatrakul, 2016). Also, confirmation can augment users’ perceived benefit and then increase perceived value in online learning (Harmandaoğlu Baz et al., 2018). Ashrafi et al. (2022) also proposed that confirmation would have positive influence on perceived value. Hence, the following hypothesis is formulated:

*H4*: PV has a positive effect on the TTF.

### Task-technology fit theory

TTF, as established by Goodhue and Thompson (1995), implies a match between the capabilities offered by technology and the requirements of a job (Dishaw and Strong, 1999) in order to determine the connection between task, technological features, and user performance (Wan et al., 2020; Sayaf et al., 2021). The TTF model has five constructs: task characteristics, technical characteristics, task technology match, utilization, and performance by MOOCs.

#### Task characteristics

Task characteristics (TCs) were utilized as mediators in earlier studies to investigate the causal correlation between the two parameters. Schulz (2005) asserts that user donations in various virtual cultures were examined using social influence as a facilitator. In a variety of studies, social status has been utilized as a mediator to reveal user intents and behaviors. For instance, Wan et al. (2020) investigated how “trust” affected how willing a user was to assent to something or somebody. The role of MOOCs links and social avoidance in affecting team cohesion was also examined by Dholakia et al. (2004) and Shiue et al. (2010). Using positive influence as a mediator, Hsiao et al. (2013) examined consumers share in virtual societies. Therefore, this study posits that task characteristics and technology characteristics are two key antecedents of students’ perceived TTF in MOOCs system for learning.

Hence, the following hypothesis is formulated:

*H5*: TAC has a positive effect on the TTF.

#### Technology characteristics

The employment of technology to create the greatest fit is referred to as “technology characteristics” (Henle and Michael, 1956). In other words, Facebook, LinkedIn, and Twitter were created with particular user groups in mind. These techniques can be employed to show how causal priming affects expected behavior (Lin and Huang, 2008). We looked into how active Twitter use encourages interaction with others and found that a number of Twitter features can help. Five mediating impacts of social media were also investigated, including the use of technologies (Facebook, Twitter, WeChat, etc.) as a platform for socializing. Media use was found to enhance task performance when task features and performance were assessed (Pal and Patra, 2021). Alraimi et al. (2015) reportedly looked at the effect of tool integration on forecasts of information and system quality. TTF and the range of performance of the team on repeated tasks were identified by MOOCs (Wang and Lin, 2011), while the TTF model is utilized to investigate the variables affecting personal performance in business resource planning (López-Nicolás and Meroño-Cerdán, 2011). They combined two distinct technological features into their frame. Hence, the following hypothesis is formulated:

*H6*: TEC has a positive effect on the TTF.

#### Intrinsic motivation

An activity is more likely to be completed by people who are strongly motivated intrinsically (Deci and Ryan, 2012). Students who are intrinsic motivation to study through MOOCs experience favorable emotions that increase their motivation to take part in such programs (Deci and Ryan, 2012; Sun et al., 2019; Badali et al., 2022). Students who are intrinsically motivated are more enthusiastic about taking MOOCs. As participation in MOOCs is governed by self-organized behavior, therefore learners should have intrinsic motivation for engaging in MOOCs. Past studies have also indicated that intrinsic motivation is more important than extrinsic motivation to indulge in self-organized behaviors. For example, Vansteenkiste et al. (2004) argued that students perform well when they are intrinsically motivated. Similarly, Lou et al. (2013) opined that individuals in online communities are strongly driven by their intrinsic motivation to share knowledge with fellow participants. It is therefore plausible to suggest that intrinsic motivation influences attitudes with regard to using MOOCs or the students’ behavioral intentions to use such an approach. Hence, the following hypotheses are formulated:

*H7*: MI has a positive effect on the ATUM.

*H8*: MI has a positive effect on the BIUM.

#### Task-technology fit

The qualities of a technology are aligned with its task features before people will adopt that technology, claims (Goodhue and Thompson, 1995) in terms of task technology fit (TTF). People may see the value of a technology, but if it is not correctly matched to the work at hand, they cannot use it to perform more efficiently (Qashou, 2021). MOOCs are frequently created so that users can efficiently complete a variety of tasks linked to learning. As a result, task-technology fit is important when it comes to investigating the acceptance of M-learning in terms of merging various viewpoints with regards to the fit based on the technology. The degree to which a system’s operational activities satisfy a person’s job needs can be taken into account when determining TTF (Goodhue and Thompson, 1995; Al-Rahmi et al., 2019b). The relationship between work requirements, individual abilities, as well as the function of the MOOCs system, is known as the TTF (Chipangura, 2019). Additionally, the relationship between TTF and the performance criteria has been established, which may be applied in the broader context of evaluating how information technology affects a person’s performance (Goodhue and Thompson, 1995; Chipangura, 2019; Al-Rahmi et al., 2019b). Hence, the following hypotheses are formulated:

*H9*: TTF has a positive effect on the MI.

*H10*: TTF has a positive effect on the ATUM.

*H11*: TTF has a positive effect on the BIUM.

#### Attitude toward using MOOCs

A person’s opinion about the introduction of new kinds of technology in any area is referred to as their ATT in the technological context (Huedo-Martínez et al., 2018). Jalil et al. (2019) assert a strong correlation between university students’ ATT toward MOOCs and their behavioral intentions (BI) to create MOOCs. According to Al-Rahmi et al. (2021b), the majority of respondents had positive attitudes when it came to using MOOC platforms, demonstrating that MOOCs are believed to be successful in enhancing conceptual knowledge throughout teaching-learning activities. According to previous research (Al-shami et al., 2022), attitude is the most effective predictor of the desire to utilize technology. Employing a Chinese sample (Luo et al., 2021), the attitude toward MOOCs and perceived behavioral control (PBC) were revealed to be important drivers of the intention to utilize them. For instance, a theoretical model based on the information system’s expectation confirmation model was proposed in another study to investigate the variables that affect MOOCs participants’ intentions to continue taking the courses (Badali et al., 2022). Hence, the following hypotheses are formulated:

*H12*: ATUM has a positive effect on the BIUM.

*H13*: ATUM has a positive effect on the SS.

*H14*: ATUM has a positive effect on the AUM.

#### Behavioral intention to use

Ajzen (2011) and Venkatesh et al. (2016) describe behavioral intention (BI) as a factor which affects a person’s purpose to adhere to and make use of a specific tool in the near future. According to Badali et al. (2022), BI was used in the majority of technology adoption studies to predict IT adoption. Ajzen (2011) asserts that BI is also believed to have an immediate impact on adoption. Studies on the uptake of MOOCs have revealed that the BI to engage in such classes has a significant impact on their actual utilization (Alraimi et al., 2015; Al-Rahmi et al., 2018; Tandon et al., 2021). Moreover. The propensity to use and keep using technology is known as Behavioral Intention to Use (BIU) MOOCs, and it is this aspect that determines how often this particular technology is used (Venkatesh et al., 2016). There is considerable support in the literature for the relationship between behavioral intention and usage behavior in general (Venkatesh et al., 2016), which has recently been extended to an MOOCs system use in higher education (Badali et al., 2022). Thus, this study examined students’ attitudes toward using MOOCs and BI of the students to do so in order to improve their learning outcomes. Hence, the following hypotheses are formulated:

*H15*: BIUM has a positive effect on the SS.

*H16*: BIUM has a positive effect on the AUM.

#### User satisfaction

User satisfaction refers to how a user feels overall when utilizing a system or application. It is based on the subjective usage judgments of the user, such as utility and efficacy (Lee, 2013). The confirmation/disconfirmation paradigm serves as the foundation for the majority of user satisfaction studies (Jacobs, 1995; Al-Rahmi et al., 2021c). A user will feel content when a system or service meets or exceeds his/her expectations. According to a number of study findings (Al-Rahmi et al., 2015b; Hong et al., 2017), user satisfaction is favorably correlated with the intention to reuse e-learning systems and MOOCs. In order to determine whether or not users would continue to utilize the e-learning system, Al-Rahmi et al., (2015c,d, 2022a) investigated key parameters. According to their findings, user perceptions in terms of content and user interface are positively correlated with users’ intentions to continue using the product. According to Wan et al. (2020), who proposed an acceptance model in the context of an e-learning service, the findings revealed that users’ intentions to continue using the service was favorably influenced by satisfaction. According to She et al. (2021), contentment is a key indicator of future MOOCs usage intentions. Hence, the following hypothesis is formulated:

*H17*: SS has a positive effect on the AUM.

#### Actual use of MOOCs

The BI to use technologies in education and the actual use of it are strongly correlated (Arpaci et al., 2020). However, the percentage of students who really use the MOOCs to study is quite low compared to their BI to do so. This may be because the MOOCs is less frequently used in academic settings than traditional approaches (Wan et al., 2020). However, through fostering social connections, peer networking, and the formation of different interpersonal bonds, it can be a useful educational tool (Fianu et al., 2018). As a result, social media sites have both good and bad effects on students, and a their behavior ultimately determines how they are affected (Al-Rahmi et al., 2019a). According to the current research, there is a connection between actual usage and student satisfaction as well as a performance impact through attitudes toward the use of MOOCs system and BI to use MOOCs.

## Research methodology

The study’s main purpose was to develop a simple and easy-to-understand theoretical model for investigating MOOCs acceptability and its factors. A multistage testing strategy was used to construct, validate, and test the suggested model and survey. To begin, participants tested 60 previously used questions to measure MOOCs acceptance at Saudi higher education, extracting 11 components to evaluate MOOCs acceptance in higher education. The questionnaire was circulated physically, then all respondents were requested to write up them to get feedback regarding MOOCs’ use for teaching and learning, also respondent’s opinion of its effect on user satisfaction and actual use of MOOCs. These questionnaires were evaluated manually and hereafter it appears obvious that12 questionnaires were not complete and thus, needed to be excluded. Thus, the rest questionnaires numbering 228 were inputted into SPSS, just to observe that 12 had some unfinished responds.

**Figure 2:**
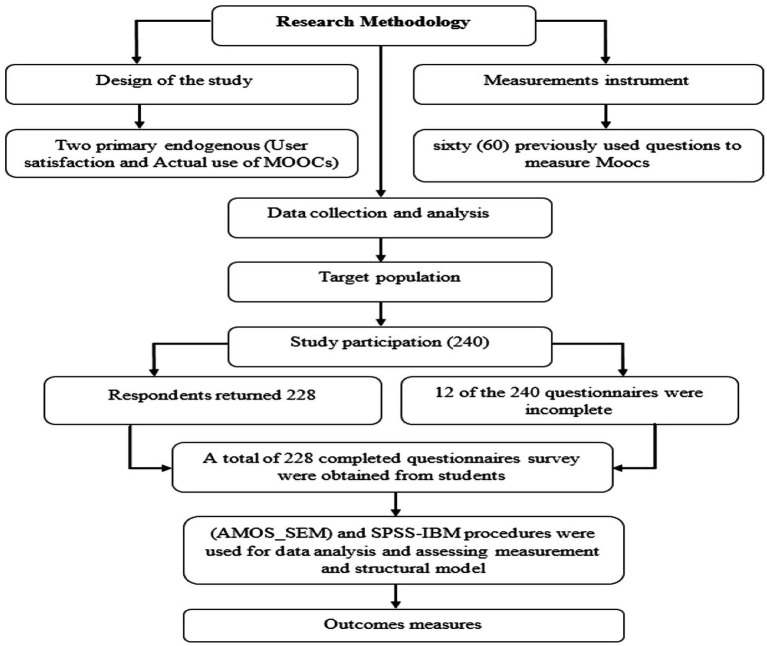
Diagram for methods and materials.

In this research, 228 questionnaire samples from King Saud University in Saudi Arabia were distributed to students throughout the summer semester of 2022, from July to August. The empirical analysis of the current study attempts to see how the interrelationships of numerous independent and dependent factors related to affective attitude toward using MOOCs, user satisfaction, and the actual use of MOOCs. Structural Equation Modeling was the statistical tool employed in the data analysis for a variety of reasons (SEM) and the diagram for research methodology (see Figure 2). The responses that have been authorized are entered into the SPSS software for analysis. This requires coding and data processing. The SPSS application is used to code the data in this investigation. The application of character symbols (mainly numerical symbols) to data is what data coding is all about. The data is modified for acceptability before being entered into SPSS and Amos-SEM. Therefore, this research received responses at 174 (76.3%) of the respondents were female, and 54 (23.7%) of them were Male. From this survey, 31 (13.6%) were in the range of 18–20 years of age, 56 (24.6%) were in the range of 21–24 years, 91 (39.9%) were in the range of 25–29 years, 42 (18.4%) were in the range of 30–34 years, and 8 (3.5%) were in the range of 35 and above. Demographic factors of specialization, 95 of the respondents from humanities with the percentage of (41.7%), 88 of the respondents from social science with the percentage of (38.6%), and 45 of the respondents from medical science with the percentage of (19.7%), see Table 1.

**Table 1:** Demographic profile.

Items	Description	*N*	%
Gender	Male	54	23.7
	Female	174	76.3
Age	18–20	31	13.6
	21–24	56	24.6
	25–29	91	39.9
	30–34	42	18.4
	35 and above	8	3.5
Specialization	Humanities	95	41.7
	Social science	88	38.6
	Medical science	45	19.7

### Measurements

A poll questionnaire was created utilizing metrics from previous literature in order to evaluate the proposed model. Every measurement item was evaluated on a Likert-type scale with five possible outcomes, ranging from one (strongly disagree) to 5 (strongly agree). Five scale items used to measure the task-technology fit were adopted from Wan et al. (2020), Yu and Yu (2010), and Wan et al. (2020), including five items measuring task characteristics and technology characteristics, while five other items were adopted from Wan et al. (2020), Yu and Yu (2010), and Wan et al. (2020). Similar to this, the scales for attitude toward using MOOCs, behavioral intentions, user satisfaction, as well as actual use of MOOCs have been derived from the studies of Wan et al. (2020) and Hew and Sharifah (2017), while the items relating to the self-determination theory (perceived connectedness, perceived autonomy, and perceived competence) were taken from Ryan and Deci (2000) and Sørebø et al. (2009). Finally, the intrinsic scale objects (five items) were taken from Wan et al. (2020) and Hernandez et al. (2011). The perceived value five-item measure was modified from Feitosa de Moura et al. (2021) and Lian and Yen (2014).

### Reliability and validity analysis

Intrinsically motivated, task-technology fit, attitudes toward utilizing MOOCs, and intent to use MOOCs were all influenced by the linked elements when using MOOCs was taken into consideration. As a result, every variable satisfied the requirements for the Cronbach alpha coefficient, which ranges from 0.70 to 0.90. The reliability analysis section discusses the Cronbach’s reliability coefficient, which in this study is 0.934 (Table 2). The study also evaluated the extent of discriminant validity in accordance with three criteria: the variable’s index value must be less than 0.80 (Hair et al., 2012), the average extracted variance rate must be greater than 0.5 (Fornell and Larcker, 1981), and the AVE square must be greater than the inter-construct correlations (IC) related factors (Fornell and Larcker, 1981; Bagozzi and Yi, 1988). Confirmatory factor loadings also met or were above the threshold of 0.7. Acceptance criteria were the Cronbach’s alpha and a composite reliability grade of 0.70 or higher (Hair et al., 2012). All of the research measures had Cronbach’s alpha values that were markedly greater than the acceptable level of 0.70 after recalibrating the item-to-total connection for all 60 items. Additionally, the coefficient alpha values also demonstrated improved trustworthiness, and the loadings of the 60 items also improved. The item-to-total correlations and the coefficient alpha (Cronbach alpha) are clearly shown in Table 3. These results support the high level of validity and acceptability of the research scales and instruments employed in this study, which will allow for additional data analysis using inferential statistics to evaluate the research hypotheses.

**Table 2:** Reliability analysis, CR, FL for the research variables.

Cod	Item	F L	CR	AVE	Cod	Item	FL	CR	AVE
Perceived relatedness	PR_1	0.792	0.915	0.682	Intrinsic motivation	IM_1	0.840	0.919	0.694
	PR_2	0.821				IM_2	0.856		
	PR_3	0.873				IM_3	0.838		
	PR_4	0.825				IM_4	0.838		
	PR_5	0.816				IM_5	0.793		
Perceived competence	PC_1	0.817	0.926	0.714	Task-technology fit	TTF_1	0.853	0.910	0.669
	PC_2	0.851				TTF_2	0.840		
	PC_3	0.861				TTF_3	0.833		
	PC_4	0.849				TTF_4	0.849		
	PC_5	0.848				TTF_5	0.704		
Perceived autonomy	PA_1	0.810	0.879	0.592	Attitude toward using MOOCs	ATUM_1	0.822	0.929	0.723
	PA_2	0.780				ATUM_2	0.834		
	PA_3	0.726				ATUM_3	0.886		
	PA_4	0.777				ATUM_4	0.852		
	PA_5	0.752				ATUM_5	0.856		
Perceived value	PV_1	0.819	0.897	0.636	Behavioral intention to use (MOOCs)	BIUM_1	0.766	0.917	0.689
	PV_2	0.807				BIUM_2	0.840		
	PV_3	0.752				BIUM_3	0.830		
	PV_4	0.769				BIUM_4	0.872		
	PV_5	0.836				BIUM_5	0.839		
Task characteristics	TAC_1	0.810	0.921	0.699	Users’ satisfaction	US_1	0.836	0.915	0.683
	TAC_2	0.778				US_2	0.877		
	TAC_3	0.859				US_3	0.872		
	TAC_4	0.891				US_4	0.793		
	TAC_5	0.839				US_5	0.748		
Technology characteristics	TEC_1	0.785	0.906	0.658	Actual use of MOOCs	AUM_1	0.831	0.930	0.727
	TEC_2	0.831				AUM_2	0.831		
	TEC_3	0.835				AUM_3	0.853		
	TEC_4	0.813				AUM_4	0.881		
	TEC_5	0.791				AUM_5	0.866		

**Table 3:** Overall validity and reliability.

	TEC	PA	TAC	PV	PC	PR	TTF	IM	ATUM	BIUM	US	AUM	Cronbach alpha
TEC	0.805												0.905
PA	0.287	0.625											0.877
TAC	0.459	0.208	0.943										0.920
PV	0.347	0.212	0.567	0.795									0.896
PC	0.454	0.236	0.434	0.415	0.838								0.926
PR	0.552	0.269	0.509	0.420	0.424	0.833							0.915
TTF	0.404	0.206	0.704	0.552	0.372	0.441	0.865						0.908
IM	0.585	0.269	0.492	0.450	0.512	0.553	0.438	0.819					0.918
ATUM	0.461	0.271	0.477	0.553	0.539	0.469	0.467	0.522	0.899				0.928
BIUM	0.491	0.256	0.486	0.437	0.509	0.478	0.417	0.530	0.483	0.790			0.916
US	0.523	0.252	0.433	0.427	0.484	0.556	0.450	0.523	0.546	0.523	0.883		0.913
AUM	0.469	0.284	0.546	0.543	0.461	0.486	0.534	0.506	0.679	0.505	0.551	0.950	0.930

## Results and analysis

### Measurement model analysis

**Figure 3:**
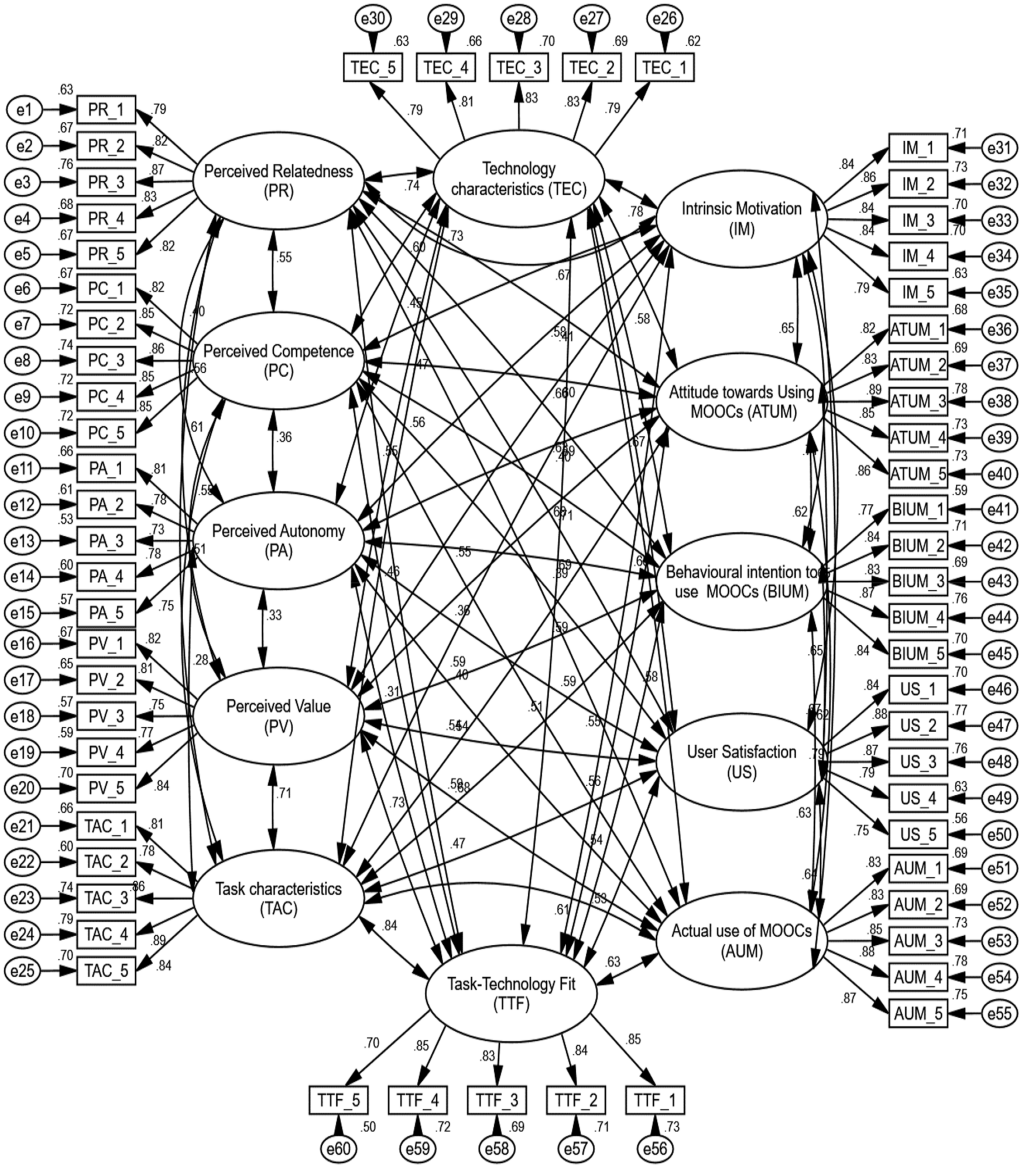
Measurement of independent, mediator, and dependent variables.

**Figure 4:**
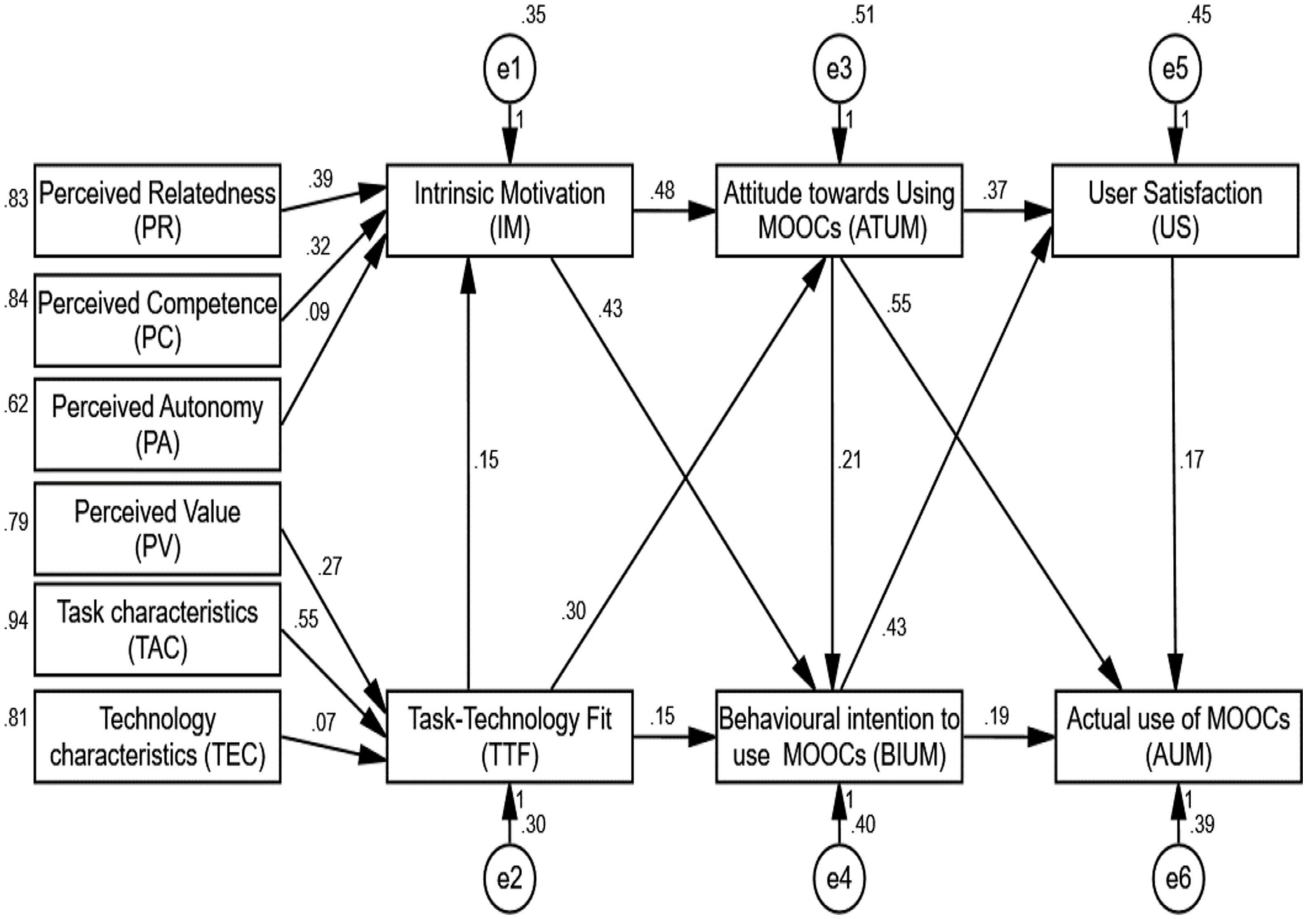
Results for the proposed model.

SEM was employed in this study as a crucial statistical technique using AMOS 23 to assess the results using confirmatory factor analysis (CFA). This model assessed over convergent while differentiating between validity, consistency, and unidimensionality (Chow and Teicher, 2003). Additionally, Cronbach’s alpha figures varied from 0.877 to 0.930, with all around 0.70, while the AVE values varied between 0.592 and 0.727, all above the predicted value of 0.50. Figure 3 displays the TTF model and SDT measurement theory. Additionally, as shown in Table 4, constructs, items, and frequentist analysis produce latent variables of 0.5 or above, which is appropriate for the response variable and evaluation of the mediator shown in Figure 4 (Fornell and Larcker, 1981; Hair et al., 2012). According to Hair et al. (2012) and Byrne (2013), the score model should also be evaluated using “goodness-of-fit” techniques including chi-square, normal Chi-square, the incremental-fit index (IFI), the relative fit index (RFI), and the Tucker-Lewis coefficient (TLI). When the normed fit value (CFI) is 0.90 or higher, the model fits well. As indicated in Table 4, the residue root mean quarter residue (RMR) is approved, and the root mean square prediction errors (RMSEA) that meets the proposed requirement as given by Hair et al. (2012) is less than and equal to 0.08 to sustain the necessary suit. The model’s suitability indices, notably CR and CA, which attest to its compliance with all standards, as well as AVE, are recognized.

**Table 4:** Goodness of fit indices for the measurement model.

Type of measure	Acceptable level of fit	Values
“Root-Mean Residual” (RMR)	Near to 0 (perfect fit)	0.39
“Incremental Fit Index” (IFI)	>0.90	0.925
“Tucker Lewis Index” (TLI)	>0.90	0.912
“Comparative Fit Index” (CFI)	>0.90	0.925
Approximation” (RMSEA)	<0.05 indicates a good fit	0.036

Table 4 presents summary out of goodness-of-fit indices applied for the assessment of the models, and Figure 3 shows the measurement of independent, mediator, and dependent variables. Figure 4 shows the all hypotheses between the 12 key constructs the 15 hypotheses were accepted, and only two hypotheses were rejected in the current sample show that students have not Perceived Autonomy leads to intrinsic motivation (0.09-H3), and also there is not relationship between Task-Technology Fit leads to Technology Characteristics (0.07-H6). Students have perceived relatedness this is leads to intrinsic motivation (0.39-H1), students have perceived competence on MOOCs and they have intrinsic motivation (0.32-H2), students have perceived value leads to Task-Technology Fit (0.27-H4). And, students have Task-Technology Fit of using MOOCs and they have Task-Technology Fit (0.55-H5), also, students’ have intrinsic motivation to Attitude toward using MOOCs (0.48-H7), Students have intrinsic motivation leads to behavioral intention to utilize MOOCs in higher education (0.43-H8), students’ have Task-Technology Fit to intrinsic motivation of using MOOCs (0.15-H9).

Similarly, student (male and female) Task-Technology Fit leads to Attitude toward using MOOCs (0.30-H10) students agreed that Task-Technology Fit improving their behavioral intention to utilize MOOCs in higher education (0.15-H11), students they have Attitude toward using MOOCs to behavioral intention to use MOOCs for learning (0.21-H12), all students have attitude toward using MOOCs leads to user satisfaction for learning through MOOCs (0.37-H13), and also, students have attitude toward using MOOCs leads to actual use of MOOCs in higher education (0.55-H14), student they have behavioral intention to use MOOCs with peers thus, improve their satisfaction (0.43-H15).

Finally, students they have behavioral intention to use MOOCs leads to actual use of MOOCs for teaching and learning (0.19-H16), and final, students (male and female) have satisfaction through MOOCs with peers thus, improve their actual use of MOOCs for learning in higher education (0.17-H17). The outcomes of the measurement model are displayed in Table 5.

**Table 5:** Structural model for hypothesis testing results.

Hypothesis	factor	Relationship	Factor	Estimate	S.E.	C.R.	*P*
H1	PR	------------>	IM	0.394	0.055	7.139	0.000
H2	PC	------------>	IM	0.320	0.052	6.200	0.000
H3	PA	------------>	IM	0.092	0.054	1.690	0.091
H4	PV	------------>	TTF	0.273	0.054	5.019	0.000
H5	TAC	------------>	TTF	0.547	0.053	10.323	0.000
H6	TEC	------------>	TTF	0.072	0.048	1.499	0.134
H7	IM	------------>	ATUM	0.478	0.061	7.781	0.000
H8	IM	------------>	BIUM	0.435	0.061	7.136	0.000
H9	TTF	------------>	IM	0.147	0.051	2.887	0.004
H10	TTF	------------>	ATUM	0.297	0.060	4.971	0.000
H11	TTF	------------>	BIUM	0.150	0.055	2.711	0.007
H12	ATUM	------------>	BIUM	0.207	0.058	3.545	0.000
H13	ATUM	------------>	US	0.373	0.057	6.504	0.000
H14	ATUM	------------>	AUM	0.549	0.058	9.498	0.000
H15	BIUM	------------>	US	0.434	0.061	7.089	0.000
H16	BIUM	------------>	AUM	0.189	0.063	3.015	0.003
H17	US	------------>	AUM	0.173	0.061	2.826	0.005

### Hypothesis testing results

The results of the hypothesis testing are shown in Table 5. The structural model and the values for each path are shown in Figure 4. (Hypothesized relationship). The hypothesized associations were evaluated using an analysis using SPSS-AMOS 23. The findings showed that perceived relatedness and perceived competence have a positive and substantial influence on intrinsic motivation (*β* = 0.394, *t* = 7.139 and *β* = 0.320, *t* = 6.200, respectively). H1 and H2 were therefore supported. H3 was disregarded since Perceived Autonomy was found to be negatively correlated with intrinsic motivation (*β* = 0.092, *t* = 1.690). On the other hand, H4 and H5 were supported since Perceived Value (*β* = 0.394, *t* = 7.139) and Task Characteristics (*β* = 0.394, *t* = 7.139) have a positive and substantial impact on Task-Technology Fit. H6 was not supported because Task-Technology Fit and Technology Characteristics were negatively correlated (*β* = 0.072, *t* = 1.499).

The attitude toward using the MOOCs system and the Intention to use the MOOCs system are both positively and significantly impacted by intrinsic motivation (*β* = 0. 478, *t* = 7.781 and *β* =0. 435, *t* = 7.136, respectively). As a result, H7 and H8 were supported. Additionally, H9, H10, and H1 were supported because Task-Technology Fit had a favorable and substantial effect on intrinsic motivation (*β* = 0. 147, *t* = 2.887), attitudes toward utilizing the (MOOCs) system (*β* = 0. 297, *t* = 4.971), and behavioral control with regard to using the MOOCs (*β* = 0. 150, *t* = 2.711). The attitude toward using the MOOCs platform has a favorable and substantial effect on users’ satisfaction (*β* = 0.373, *t* = 6.504), behavioral intent to use the (MOOCs) system, and actual usage of the (MOOCs) system (*β* = 0.549, *t* = 9.548). H12, H13, and H4 were therefore supported. The users’ satisfaction (*β* = 0.434, *t* = 7.089) or actual usage of the MOOCs system (*β* = 0.189, *t* = 3.015) are also positively and significantly impacted by behavior intent to use the (MOOCs) system. H15 and H16 were thereby supported. Last but not least, the findings showed that users’ contentment has a favorable and substantial impact on the actual use of the MOOCs system (*β* = 0. 173, *t* = 2.826). H17 was therefore supported.

## Discussion and implications

The purpose of this study is to examine the relationship between independent variables namely, Perceived Relatedness, Perceived Competence, Perceived Autonomy, Perceived Value, Task Characteristics, Technology Characteristics with Intrinsic Motivation and Task-Technology Fit, and the mediating Intrinsic Motivation, Task-Technology Fit, and Attitude toward using the MOOCs system, Behavioral intention to use MOOCs system with User Satisfaction and Actual use of MOOCs system in higher education for Saudi academic student.

By providing a competitive alternative to traditional classroom education through ICT and connectivity, MOOCs have changed teaching and learning. Through the use of interactive multimedia and the digital environment, MOOCs encourage students to collaborate on their learning in novel ways. MOOCs are a useful alternative to the traditional classroom model of learning given the high expense of education as well as the issue of accessibility. In terms of further investigation and application, the current study provides important conclusions. According to the study’s findings, which are in line with those of Moafa et al. (2018), Aldowah et al. (2020), Vanduhe et al. (2020), and Wan et al. (2020), task and technological characteristics help to achieve a task-technology fit that positively influences behavioral intention, indicating that MOOCs are suitably matched to students’ learning tasks. The study also looks into societal issues that influence MOOCs acceptance. Perceived relatedness and Perceived Competence (H1 and H2) has been shown to positively influence students’ Intrinsic Motivation, which suggests that it can improve social interaction and increase the acceptance of MOOCs. In contrast to other research, this study discovered that social impact had no meaningful association with the Intrinsic Motivation of students, indicating that internal pressures are more important than external ones for the uptake of MOOCs (Luo et al., 2021). The lack of robust social engagement opportunities when using MOOCs could be the cause. According to the findings, perceived connectedness has a favorable impact on students’ inclinations to enroll in MOOCs, supporting the previous findings of Hew and Sharifah (2017). This indicates that students like to be associated with the top universities and instructors. Using SDT and TTF as the conceptual approaches, the aim of the current study was to analyze the factors behind the acceptance of MOOCs. We looked into the learners’ intention to use MOOCs from three angles: task features, technology characteristics, or task-technology fit, based on TTF (Al-Rahmi et al., 2022b). The findings showed that SDT and TTF environment had a substantial impact on the desire to use MOOCs. The SDT factors, we had taken into account related to relatedness, self-efficacy, and perceived autonomy. Each of these factors were determined to have negligible effects. This shows that even if students have the technological and financial means to use MOOCs, learning through them is not necessarily the goal. This result is consistent with some earlier research that indicated the negligible effect of TTF on students’ use of MOOCs (Alyoussef, 2021; Shanshan and Wenfei, 2022) and accepting behavior (Mohan et al., 2020). Being Perceived Autonomy (H3) has been shown to do positively influence students’ Intrinsic Motivation, which suggests that it can improve social interaction and increase the acceptance of MOOCs. This result conflicts with findings of Ma and Lee (2019) and Lung-Guang (2019), each of whom discovered a notable favorable effect of abilities on the intrinsic motivation for real MOOC use. Although a consideration of perceived autonomy has not been able to pinpoint the inner drive for actually using MOOCs, it may turn out to be a key factor (Alonso-Mencía et al., 2020), which has not been included in the current study.

According to perceive value (H4) is indeed the second crucial factor in determining Task-Technology Fit and practical use of MOOCs. This result is consistent with that of Ma and Lee (2019), who discovered that the performance-to-cost value of MOOC utilization had a substantial impact on task-technology fit. This suggests that students will be motivated to participate in MOOCs if they believe they would be worth paying for (Al-Rahmi et al., 2015a; Al-Maatouk et al., 2020; Feitosa de Moura et al., 2021). If people believe that the value acquired through MOOCs in terms of skill improvement or academic performance improvement justifies investing money, time, and effort, they will be willing to embrace MOOCs. The conclusion might potentially be a result of educators’ and students’ skepticism regarding the accessibility of the different software or hardware requirements for MOOCs use. The investigation also revealed two new pathways: Task Characteristics and Technology Characteristics (H5 andH6). These two aspects have an immediate impact on the Task-Characteristics Compatibility for using MOOCs going forward. It is suggested that whether or not MOOCs are useful would depend on the types of tasks that consumers are engaged in. Strong correlations between task Characteristics and TTF (H5) compatibility have been found by Moafa et al. (2018), Wan et al. (2020), and Alyoussef (2021). Based on the outcomes of the AMOS analysis, the variable Technology Attributes was selected as a factor that might potentially affect how consumers use MOOCs. Additionally, this study found a negative correlation between Task-Technology Fit and Technology Characteristics (H6) for users’ adoption of MOOCs. The aspects of MOOCs that are important for task completion are reflected in the Technology Characteristics. It has been demonstrated that Task Characteristics have an impact on a system’s utility, simplicity of use, precision, adaptability, and reliability (DeLone and McLean, 1992). According to Moafa et al. (2018), Wan et al. (2020), and Alyoussef (2021), significant associations among Task Characteristics with TTF have been found.

Students’ Intrinsic Motivation was reported to positively influence Attitude toward using (MOOCs) system (H7), Behavioral intention to use (MOOCs) system (H8) in user satisfaction and actual use of massive open online courses (MOOCs) systems. It is discovered that intrinsic motivation has a significant impact on both behavioral intentions to utilize MOOCs, and attitude toward utilizing MOOCs. Students are drawn to and willing to enroll in MOOCs when they believe that learning through them is entertaining and pleasurable. This hypothesis explains the previous studies provided by Shahzad et al. (2020) and Abdullatif and Velázquez-Iturbide (2020). Students in poor nations like Saudi Arabia experience significant stress and hardship as a result of the old teaching and learning system. Given that there are no set completion dates for these courses, students are drawn to the MOOCs learning environment since they can work them at their own pace. Additionally, MOOCs offer reusable learning materials in the form of educational videos that students can refer to in the future. These aspects encourage students to participate in MOOCs for learning. The SDT is seen to play a significant role in influencing learners’ satisfaction and practical use of MOOCs, which is in conformity with the results of Khan et al. (2018) and Sun et al. (2019). The motivation for using MOOCs is created when all three fundamental psychological needs the need for relatedness, the need for competence, and the need for autonomy are met. This intrinsic motivation in turn affects learners’ attitudes toward using MOOCs, their behavioral intentions to use them, and their actual use of MOOCs throughout higher education. Students become intrinsically motivated to engage with MOOCs when they believe they can control their learning through them, maintain connections with others, and feel qualified while studying (Al-Rahmi et al., 2018; Sun et al., 2019; Al-Rahmi et al., 2021b). The findings indicate that Task-Technology Fit is a strong predictor of intrinsic Motivation of students (H9), Attitude toward using (MOOCs) system (H10), and Behavioral intention to use (MOOCs) system (H11) in that the more MOOCs are Technology Fit, the more likely students will be to perceive the MOOCs as useful. This result is consistent with previous studies (Abdullah and Ward, 2016). Specifically, the direct effect of Task-Technology Fit on attitude toward using of MOOCs was found to be apparent. In addition to mediating factors all other criteria are found to have substantial beneficial effects on users’ satisfaction and their actual usage of MOOCs in the context of actual use of MOOCs, with the exception of Attitude toward the use of MOOCs and Intention to use the MOOCs system (H12, H13, H14, H15, H16, H17). According to research by Khan et al. (2018), Alyoussef (2021), Al-Adwan et al. (2021), and Senevirathne et al. (2022), the strongest predictor of users’ satisfaction and actual use of the MOOCs system is their attitude toward using them and their behavioral intention to use. This shows that the most important motivator for students when it comes to actually using MOOCs is user satisfaction and the system of MOOCs certification, even in the case of research universities and employers.

### Theoretical and practical implications

According to the study’s findings, intrinsic Motivation, task-technology fit, attitude toward utilizing MOOCs, and behavioral intention for using MOOCs all play significant roles in determining users’ satisfaction with, and actual use of MOOCs. This has various beneficial applications in real life. First, since perceived relatedness, perceived Competence, and perceived autonomy are the foundations of intrinsic motivation, self-paced courses from prominent universities should be a priority for MOOCs providers. Even though the majority of MOOCs are self-paced, some nevertheless have set start and end dates for their courses. Self-paced courses are more frequently chosen by students than those with set lengths of time.

The providers of MOOCs should also promote participatory learning, which enables students to connect with other participants by showcasing their educational objectives and imparting knowledge in blogs or online forums. Second, academic institutions should offer credit benefits to students as an alternative to having those complete courses through MOOCs, given the significance of TTF. This will motivate students to participate in MOOCs. Employers ought to acknowledge MOOCs diplomas and offer credit where credit is due to students who successfully complete them. Third, given the importance of perceived value, MOOCs providers should concentrate on offering affordable courses in a variety of cutting-edge disciplines. Paying significant sums for MOOCs certification is challenging, particularly in underdeveloped nations where students face financial and educational resource limits.

## Conclusion and future work

Finally, SDT and TTF were included in the updated model for assessing MOOCs usage and acceptance in educational institutions. The current study filled a gap in the literature by examining the relationships between perceived relatedness, self-efficacy, perception, autonomy, potential value, task identity, and technology characteristics, as well as the importance of intrinsically motivated, TTF, attitudes toward using MOOCs, and behavioral intention for using MOOCs as an intermediary of these relationships. The SEM-Amos research suggests that user perception (PR, PC, and PA) can have a favorable impact on IM’s propensity to use MOOCs. Additionally, TTF was well predicted by TEC, PV, and TAC. The research’s conclusions show that the model may be used to understand the effects of TTF, ATUM, BIUM, users’ satisfaction, and the context of actual MOOCs use. The model’s applicability to both lecturers and students shows that it is generalizable across these sub-samples, indicating that it is pertinent to both students’ learning and the professional growth of academics.

First of all, learners’ reasons for taking part in MOOCs differed, with university students appearing to be more driven by the desire to further their education. Second, learners with autonomous and controlled observed increased scores for course design, interplay with teachers and peers, perception relatedness, Perceived Competence, perceived autonomy, attitude toward using the MOOCs, behavioral actual intent to use the MOOCs, users’ satisfaction, as well as actual use of the MOOCs when compared to learners with governed and combined motivation groups. Third, there were many TTF variables that were significantly-and favorably-related to users’ pleasure and actual usage of the MOOCs, including attitude toward using the system and behavioral intention to utilize it. Fourth, the effects of users’ satisfaction and actual usage of the MOOCs were favorably and significantly boosted by intrinsic motivation, TTF, attitudes toward using the MOOCs, and behavioral control to utilize the system. The sample size of the study was restricted with one university in Saudi Arabia, which is one of the limits of the study, despite the fact that it produced some surprising results. The effectiveness of private universities, or educators may not be revealed by the outcomes. The fact that this study exclusively employs questionnaires is one of its other shortcomings. The study lacks qualitative information and is based solely on students’ perceptions, which may differ from teachers’ perceptions. The research does not take differences between research disciplines into account either. It is advised that future research seek to obtain findings in nations with different values and more accurately account for these additional restrictions.

## Data availability statement

The original contributions presented in the study are included in the article/Supplementary material, further inquiries can be directed to the corresponding author.

## Ethics statement

Ethical review and approval was not required for the study on human participants in accordance with the local legislation and institutional requirements. Written informed consent from the patients/participants or patients/participants legal guardian/next of kin was not required to participate in this study in accordance with the national legislation and the institutional requirements.

## Author contributions

UA and AA: conceptualization, methodology, validation, resources, data curation, writing—original draft preparation, writing review and editing, and funding acquisition. UA: software, investigation, supervision, and project administration. AA: formal analysis and visualization. All authors contributed to the article and approved the submitted version.

## Funding

This research was supported by Researchers Supporting Project number (RSP2023R159), King Saud University, Riyadh, Saudi Arabia.

## Conflict of interest

The authors declare that the research was conducted in the absence of any commercial or financial relationships that could be construed as a potential conflict of interest.

## Publisher’s note

All claims expressed in this article are solely those of the authors and do not necessarily represent those of their affiliated organizations, or those of the publisher, the editors and the reviewers. Any product that may be evaluated in this article, or claim that may be made by its manufacturer, is not guaranteed or endorsed by the publisher.
