# Supplementary material for: Integrated TTF and self-determination theories in higher education: The role of actual use of the massive open online courses
Source: Front Psychol. 2023 Feb 2;14:1108325. doi: 10.3389/fpsyg.2023.1108325 (PMC9933983; doi:10.3389/fpsyg.2023.1108325)
Supplement: Supplementary file 1 [file Table_1.docx]

**Appendix**

| Perceived Relatedness (PR) | | | |
| --- | --- | --- | --- |
| 1. | | | I pretty much keep to myself when I am at work. |
| 2. | | | I get along with people at work. |
| 3. | | | I consider the people I work with to be my friends |
| 4. | | | The people I work with do not seem to like me much. |
| 5. | | | People at work are pretty friendly towards me. |
| Perceived autonomy (PA) | | | |
| 6. | | I feel like I can make a lot of inputs to deciding how I learn the course. | |
| 7. | | I feel like I can pretty much be myself when learning in MOOC system. | |
| 8. | | There are many opportunities for me to decide for myself what and how I learn in MOOC system. | |
| 9. | | I can make my own decisions about MOOC system learning | |
| 10. | | I am free to express my ideas and opinions on using MOOCs in my educational work | |
| Perceived Competence (PC) | | | |
| 11. | I do not feel very competent when I use MOOCs in my educational work. | | |
| 12. | The other faculty tell me I am good at using MOOCs in my educational work. | | |
| 13. | I have been able to learn interesting new skills in MOOCs through my job. | | |
| 14. | Most days I feel a sense of accomplishment from working with MOOCs. | | |
| 15. | When I am using MOOCs I often do not feel very capable. | | |
| Intrinsic Motivation (IM) | | | |
| 16. | I find participating in MOOC system to be enjoyable. | | |
| 17. | The actual process of participating in MOOC system is Pleasant. | | |
| 18. | I have fun participating in MOOC system. | | |
| 19. | I do not realize the time elapsed when using MOOC. | | |
| 20. | Use of MOOC stimulates my curiosity | | |
| Perceived Value(PV) | | | |
| 21. | MOOCs are reasonably priced. | | |
| 22. | Learning through MOOCs is value for money. | | |
| 23. | It is worth spending time in MOOCs. | | |
| 24. | Using MOOCs optimizes the study time | | |
| 25. | MOOCs are perfectly and precisely the resource needed for the teaching-learning process. | | |
| Task-Technology Fit (TTF) | | | |
| 26. | MOOCs are fit for my learning requirements. | | |
| 27. | Using MOOCs fits with my educational practice. | | |
| 28. | It is easy to understand which tool to use in MOOCs. | | |
| 29. | MOOCs are suitable for helping me complete online courses. | | |
| 30. | I think that using MOOC is well suited for the way to learn. | | |
| Task characteristics (TAC) | | | |
| 31. | I need to acquire online learning resources related to the courses I study. | | |
| 32. | I need to access MOOCs conveniently and quickly | | |
| 33. | The contents of MOOCs should be of high quality | | |
| 34. | The contents of the online courses should meet my learning requirement. | | |
| 35. | I often need to communicate with other classmates to improve my learning. | | |
| Technology characteristics (TEC) | | | |
| 36. | MOOC platform has the function of resource acquisition and sharing. | | |
| 37. | MOOC platform provides social interaction services. | | |
| 38. | MOOC platform provide high-quality learning materials. | | |
| 39. | MOOC platform is convenient to access through various mobile devices. | | |
| 40. | MOOC platform provides multiple evaluation functions. | | |
| Attitude towards using (MOOCs) system (ATUM) | | | |
| 41. | I believe that using MOOCs is a good idea. | | |
| 42. | I believe that using MOOCs is advisable. | | |
| 43. | I am satisfied with using MOOCs. | | |
| 44. | Studying is more interesting with MOOCs | | |
| 45. | I am happy when I am able to answer the practice questions in the MOOC. | | |
| Behavioural intention to use (MOOCs) system (BIUM) | | | |
| 46. | I intend to continue to use MOOCs for learning new concepts in the future. | | |
| 47. | I predict that I would use MOOCs for sharing learning materials in the future. | | |
| 48. | I predict that I would frequently use MOOCs to watch video lectures in the future. | | |
| 49. | My overall intention to use MOOCs in the future is very high. | | |
| 50. | I will insist on using MOOCs to study the courses I registered for. | | |
| User Satisfaction (US) | | | |
| 51. | I would be satisfied with my decision to undertake a MOOC. | | |
| 52. | If I had the chance to undertake a MOOC, I would be delighted to do so. | | |
| 53. | I would be very satisfied with a MOOC. | | |
| 54. | I feel that MOOCs are well-suited to my needs. | | |
| 55. | I will undertake as many MOOCs as I can. | | |
| Actual use of MOOCs system (AUM) | | | |
| 56. | I often use MOOCs to manage my tasks | | |
| 57. | I usually use MOOCs. | | |
| 58. | I regularly use MOOCs. | | |
| 59. | I frequently complete courses from a MOOC site. | | |
| 60. | I use MOOCs for academic purposes to discuss and share my ideas with my peers | | |
